# When Common Birds Became Rare: Historical Records Shed Light on Long-Term Responses of Bird Communities to Global Change in the Largest Wetland of France

**DOI:** 10.1371/journal.pone.0165542

**Published:** 2016-11-10

**Authors:** Thomas Galewski, Vincent Devictor

**Affiliations:** 1 Institut de recherche de la Tour du Valat. Le Sambuc, 13200 Arles, France; 2 Institut des Sciences de l'Evolution, UMR 5554, Université Montpellier 2, 34090 Montpellier, France; Consejo Superior de Investigaciones Cientificas, SPAIN

## Abstract

Many species have suffered large population declines due to the anthropogenic influence on ecosystems. Understanding historical population trends is essential for informing best efforts to preserve species. We propose a new method to reconstruct the past structure of a regional species pool, based on historical naturalist literature. Qualitative information collected from annotated checklists and reports can be relevant to identify major long-term community changes. We reviewed ornithological literature on the Camargue, the largest wetland in France. We reconstructed the entire breeding bird community from 1830 to 2009 and translated historical data into semi-quantitative data. This data permitted a calculation of a Community Commonness Index to measure the average level of abundance of species in a community. The Community Specialization and Community Temperature Indices were used to evaluate the potential long-term impact of land-use and climate changes on the composition of the regional bird species pool. We found a decrease in average abundance and specialization between 1950 and 1989, suggesting that changes in land-use negatively impacted the structure and composition of the local bird community by reducing species abundance and removing habitat-specialists (e.g. Southern Grey Shrike, Greater Short-toed Lark). These results are likely to be linked with a major loss of natural habitats in the Camargue between 1942 and 1984 when natural areas and traditional farmland were converted into intensive cultivated lands. We also found fluctuations among species with high versus low temperature preference. However, long-term effects of climate change on the bird community might be blurred by the impact of land-use changes. Overall, our results contrast with those obtained from well-monitored colonial waterbirds showing long-term increases. Our results plead for a more regular use of historical naturalist data when examining long-term changes in species communities as they allow the establishment of an older temporal point of reference and consideration of species not covered by traditional monitoring schemes.

## Introduction

In conservation, identification of time periods of severe population decline is critical to determine potential threats. If the causes of decline are not understood, it is difficult to create conditions to promote population recovery [1]. Unfortunately, population monitoring often only begins after declines are evident [2]. Monitoring protocols may also have changed over time, making comparisons between the present and the past difficult [3].

Bird populations are among the best monitored animal populations [4,5]. Data collected in long-term monitoring schemes are increasingly used to calculate multi-species indices and explore the driving forces of changes in bird populations and their habitats [6–8]. In Europe, long-running surveys were first initiated at the beginning of the twentieth century and concentrated on colonial species, such as breeding herons and seabirds [9,10]. Large-scale breeding bird surveys of more common species were not implemented until the 1960s in the United Kingdom, and not before 1990 and 2000 in most other European countries [7,11–13]. The two last decades are therefore generally used as points of reference when inferring species trends [14–16]. This is problematic because by that time, bird populations had already responded to large-scale changes in the European landscape, such as industrialization and agriculture intensification [17–19]. A major risk emerging from this lack of knowledge is the underestimation of the historical decline of some species or, conversely, magnification of recent increases in population size that actually remain far below their historical level. For instance, the Ortolan Bunting is a common bird which has experienced a recent large decline (estimated to have declined in France by 51% since 2001 http://vigienature.mnhn.fr/page/bruant-ortolan). However literature shows a major and much earlier contraction of its breeding range between 1950 and 1970.

Some authors advocated for combining different approaches for the evaluation of population trends [2]. They suggested that traditional approaches based on census data which are often short term in perspective should be complemented by non-traditional approaches like using pre-existing data gathered from the past, from documents such as naturalists’ field notes. Using information from such field notes, allows to include species usually not taken into account by traditional monitoring schemes because of their discrete habits or scarcity [20]. In the absence of long-term quantitative data spanning the entire species communities, the use ofnon-scientific naturalist literature may therefore provide key information to understanding population changes at the community level.

To understand the fate of species communities over time, it is important to have both an idea of trends in species richness and community structure (i.e. species occurrence and relative abundances) and composition [21]. Even in stable environments, one expects to observe fluctuations in community composition due to extinction and colonization events. However, current global changes can lead to non-random changes in community composition if species with specific traits are filtered out or favored by those changes.

Among the various anthropogenic factors that drive the decline of biodiversity, two are considered of major importance: climatic change [22] and the destruction, fragmentation and disturbance of habitats often referred to as land-use change [23,24]. Indicators accounting for species-specific responses to land-use or temperature change were recently developed and applied at the European or national level to evaluate the response of bird communities over time [25–27].

The relevance of these indicators rests on the fact that each species may react differently to global changes. They for instance capture that bird species with no strong habitat requirements (habitat-generalists), are better able to survive the modification of ecosystems than species tightly linked to few habitats (habitat-specialists), leading to a higher representation at community level. Simultaneously, with the general increase in temperatures, one should expect the relative abundance of hot-dwelling species in the community to increase over time. Although these indicators were successfully used with standardized monitoring[25–27], it is unclear whether they could be used to capture longer term changes in community composition using heterogeneous records of species occurrence. Overall, historical changes in community structure and composition are hardly documented or only for very local areas (most often in island landscapes) [28].

**Fig 1:**
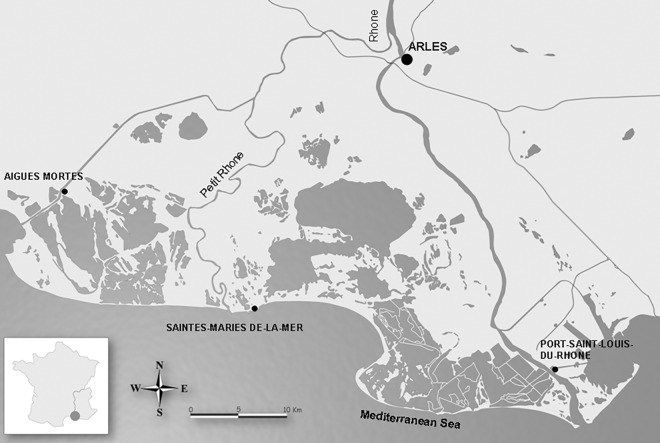
Localization and map of the Camargue.

The Rhone delta or Camargue (Southern France; Fig 1) is one of the most extensive wetlands in Europe (180.000 hectares) and harbors a diversity of habitats which sustain more than 400 bird species [29]. Large part of the European waterbird populations breed in the Camargue [30] which motivated the designation of several nature reserves, Ramsar sites and a Biosphere Reserve. The Camargue has been a famous birdwatching site since the nineteenth century [31], providing abundant qualitative information on the status of all recorded bird species. As many wetlands, the Camargue has been largely impacted by the development of human activities [32]. Like other Mediterranean deltas [33], the water cycle was almost completely controlled by the erection of hundreds of kilometers of dykes during the nineteenth century to prevent seasonal flooding of the Rhone River. Securing the area from flooding allowed for the development of large-scale agriculture and some industries at the expense of large areas of natural habitats. Moreover, the Camargue has experienced an increase in the mean annual temperature between 1960 and 2010 (around 1°C; Tour du Valat data). It is suspected that these changes have had an impact on the bird community although their relative contribution is unknown. Given this context and history, the Camargue is a representative case study for identification of the long-term impacts of land use and climate change on bird communities for other wetlands and lowland areas, providing useful information for sites where data on birds does not exist for such a long time period.

In this manuscript, we propose and apply a new method to reconstruct the past structure of a regional species pool, based on historical naturalist literature, using historical and contemporary ornithological literature to assess the species richness and relative abundance in the Camargue over the last 180 years. Based on climatic data across their whole range, we characterized each species by their temperature affinity as well as by their level of habitat-specialization to test whether directional shift in community composition could be observed from a long-term response to global changes.

## Materials and Methods

### Data origin

The avifauna of the Camargue has been studied for a long time by local naturalists with the first inventories initiated in the eighteenth century [34], and the first comprehensive annotated checklists published in the nineteenth century [31,35]. At the beginning of the twentieth century, foreign birdwatchers started to regularly visit the area, allowing the publication in 1930s of new checklists with updated status and abundance [36,37]. The Tour du Valat biological station, founded in 1954, allowed the installation of ornithologists in the heart of the Camargue year-round. Reports have since been published every few years to synthesize the salient observations and report significant changes in the local status of bird species [29,38–45]. In addition, three comprehensive checklists of birds of the Camargue were published in the last thirty years [46–48]. Nowadays, bird records are most commonly made by professional ornithologists from diverse organizations (i.e. Tour du Valat, Conservatoire des Espaces Naturels, Association des Amis des Marais du Vigueirat, Centre National de la Recherche Scientifique, Centre Ornithologique du Gard, Conservatoire du Littoral, Ligue de la Protection des Oiseaux, Pont de Gau, Office National de la Chasse et de la Faune Sauvage, Société Nationale de la Protection de la Nature, Syndicat mixte de Camargue gardoise) as well as by many birdwatchers who submit their observations to the above-mentioned organizations.

### Reconstruction of the breeding bird community over time and quantification of species commonness

First, we constructed a presence-absence matrix reporting the breeding status of all bird species over time. For each species we indicated if the breeding was known for each of eight time periods: around 1830 (1820–1840), around 1930 (1920–1936), then the last six decades 1950–59, 1960–69, 1970–79, 1980–89, 1990–99 and 2000–09. Data did not allow to reconstruct the breeding bird community for the 1940–49 time period due to reduced birdwatching activity during World War II. The reconstitution of the breeding bird community within ten-year periods over the 1950–2009 period required consultation of consecutive reports to obtain information on all species.

Obviously the use of historical naturalist data has some biases. First, the older ornithological literature usually dealt with a geographical area slightly larger than the present-day extent of the Camargue. However, we assume that birds breeding in low coastal plains bordering the Camargue were also present in the delta. Second, we had to trust previous species identification, despite that knowledge on bird identification was less accurate than today. Except for rare cases where nesting in the Camargue seems highly unlikely and was not sufficiently documented (e.g. Green Sandpiper or Willow Warbler), we assumed that observations were nevertheless correct. However, the knowledge of local avifauna by historical birdwatchers was often outstanding as suggested by their very precise observations, often based on specimens and eggs collected in the field. Third, birdwatching intensity considerably varied over time showing an increase of reported sightings. Consequently, the number of breeding species was probably underestimated for the older periods. While this impacts the precise quantitative estimates and create periodic increase of species richness, this bias should not affect the qualitative results regarding changes in species composition and decrease of particular species.

From all the collected data, we built a presence-absence matrix of each species’ presence or absence for each time period. In most ornithological checklists and reports consulted, authors provided an assessment of the level of abundance per species. We therefore also constructed a relative abundance index by distinguishing rare from common breeders. We considered a species as “rare” when the authors stated that it bred irregularly, or in low numbers and/or was highly localized in the study area. When estimates of population numbers were given, a bird species was referred to as “rare” when there were fewer than 100 breeding pairs and” common” with a breeding population estimated to be higher than 100 pairs. This resulted in a matrix where we attributed a “Species commonness index” (SCI) for each species and for each time period. There were three possible values: “0” when breeding was unknown, “1” for rare breeders and “2” for common breeders (S1 Table). By using this coding, only large detected increases or declines were accounted for; however, the advantage was to limit subjectivity of birdwatchers. We also submitted the matrix to three ornithologists who have solid and long-lasting knowledge in Camargue avifauna for both recent (1990s-2000s) and historical periods (1960s-1980s), in order to point out potential disagreements on species statuses. When contradictions were found, we returned to the literature for further information.

To characterize the average abundance of a species in the overall bird community (i.e. the entire species pool), a Community Commonness Index (CCI) was calculated for each time period using a community weighted mean given by CCI = Σa_*i*_/Σ *i*, where *a*_*i*_ designates the abundance of species *i* in the bird community at that time (note that *a* = 0, 1, or 2 as defined previously). If a species pool contains more species improving in status (from 0 to 1, or 0 to 2, or 1 to 2) than species declining (from 2 to 1, 2 to 0, or 1 to 0) from time *t* to *t*+1, then CCI_*t*_ of this species pool increases from time *t* to *t*+1,

CCI was calculated for the entire regional species pool, but we also ran separate analyses for main habitat types found in the delta. A classification of common bird species to habitat types exists for Europe [49] and is continuously updated by the European Bird Census Council (EBCC). Each bird species recorded as a breeder in the Camargue was classified according to the 2015 update for the Mediterranean region (http://www.ebcc.info/index.php?ID=592#Box%20Species%20selection%20and%20classification). Using this list and the habitat types present in the Camargue, we distinguished three different habitat specialists categories for birds: ““farmland”, “forest” and “wetlands (S2 Table). Only wetland (n = 69) and farmland (n = 53) species were taken into account for analyses about habitat specialists. The forest species pool (n = 20) was too small and the remaining “other” species did not provide any information on the habitats used by birds.

### Community Specialization Index and Community Temperature Index

We calculated two community weighted means to reflect changes in community composition driven by land-use or climate changes [25,26]. For these two indices, *X*_*i*_, is attributed to each species *i* reflecting the specific sensitivity to a given pressure (land-use or climate change). In doing so, each species can be ranked along a continuous gradient from the least sensitive to the most sensitive species for a given pressure. Then, any given species pool at a given point *t* in time can be characterized by averaging trait *X*_*i*_ across individuals present in this species pool at that time. These community level indices are simply a weighted average given by CXI_*t*_ = Σ(*a*_*i*_*X*_*i*_)/Σ*a*_*i*_, where *a*_*i*_ designates the abundances of species *i* in this species pool and *X*_*i*_ the specific trait of species *i*. Then, if this species pool, which is characterized by CXI_*t*_ is affected by the given pressure of interest from time *t* to *t*+1, each species should adjust its abundance according to its sensitivity to that pressure. This would result in a new value of CXI_*t*+1_ which is different from CXI_*t*_ and which mirrors the average change of each species-specific response to that pressure in this assemblage. Typically, following an increase of a given pressure to which a species is more or less sensitive according to their *X*_*i*_, the species with high *X*_*i*_ should increase relatively faster than those with low *X*_*i*_ so that CXI should increase from t to t+1. Conversely if the pressure decreases, CXI should decrease and remain stable on average if the pressure stays constant.

To track land-use change impacts on communities, a species-specific level of specialization was developed as a proxy for *X*_*i*_, the Species Specialization Index (SSI) [25]. As specialists are expected to be replaced by generalists following habitat loss or disturbance, the Community Specialization Index is therefore calculated as CSI = Σ(*a*_*i*_SSI_*i*_)/Σ*a*_*i*_ and should decrease following landscape modification. Similarly, this approach was used to quantify climate change impacts on communities. In this case, *X*_*i*_ was replaced by the Species Temperature Index (STI). STI of a given species is simply the average temperature of the species' breeding season range. Following temperature increases, one expects species with breeding areas characterized by high average temperatures (i.e., with high STI) to replace those breeding in colder ranges. Therefore, the CTI given by CTI = Σ(*a*_*i*_STI_*i*_)/Σ*a*_*i*_ is expected to increase following climate warming [26].

We used the SSI values from Leviol et al. [50], where the SSI is the variation in bird species preference for different EUNIS habitat types [51] (S1 Table). Habitat preference describes a species’ affinity level for nesting and foraging per EUNIS habitat. Levels of affinity are coded from 1 to 3 by experts to describe increasing habitat preference (code 3: primary habitats; 2: secondary; 1: others). The higher level was retained per species for each habitat and SSI values were calculated as the coefficient of variation of species affinity across the range of habitat classes. The coefficient of variation was used as the metric since it is statistically independent of the average species density [52]. We calculated the CSI of the entire Camargue breeding bird community by averaging the SSI of the constituent species weighed by their index of commonness (0, 1, 2), as a proxy for their relative abundance. The CSI was calculated for each of the eight time-periods defined above.

STI—the long-term average temperature experienced by the individuals of a species over its range in the breeding season (S1 Table)—was determined for each species using distributional data from the European Bird Census Council atlas of European breeding birds [53] as well as patterns of mean annual temperatures across Europe from the WorldClim database (URL http://www.worldclim.org) [26]. For any given species pool, the CTI was calculated by averaging the STI of the constituent species as described for the CSI. Note that, although only a part of each species’ range was considered, the CTI based on European climate data can be applied due to the high correlation between continentally and regionally determined STI [26]. Species with higher STIs are those breeding in warmer ranges on average. Since these species are also those breeding in southern latitudes, an increase in CTI can be viewed as reflecting the replacement of northerly distributed species by southern species. Note that the SSI and STI of the Camargue breeding bird species are uncorrelated (*r*_*S*_ = 0.030, *p* = 0.714), thus the trend in CSI and CTI can be considered as two independent aspects of community change.

### Data analysis

We first estimated the linear trends in each community indices (CCI, CSI and CTI) through time accounting for temporal autocorrelation. We fitted General Least Square (GLS) models with year as a predictor and an autoregressive term of order 1 [54]. Note that in this analysis, we had only one value for each index in each time period. We therefore did not take into account potential sources of within-year uncertainty of each index. It is possible that part of the observed yearly fluctuation in each index (if any) could simply result from a fluctuation in species richness. For instance, if rare and common species were not uniformly distributed, increasing species richness–a possible consequence of the increase in survey effort over time—could increase the probability to record a rare species therefore spuriously decreasing CCI. Similarly, depending on how species with high or low sensitivity to land-use or climate changes (i.e with different values of SSI or STI) are distributed among species-rich or species-poor assemblages, an increase in CSI or CTI could simply reflect a change in species richness.

We further tested the robustness of the decadal change in each community index to change in the accuracy of the exact set of species recorded in each time period. This analysis was conducted to assess the expected value of each index following a random fluctuation of species identity among the whole avifauna recorded in the study area. To do so, we calculated for a given index and a given period 1000 values of the expected index calculated with the same number of species recorded that given year but randomly sampled in the total species pool. If directional trends in a given index are observed one should expect a marked difference between the yearly index calculated with the observed species and those calculated with random species subsets.

All statistical analyses were carried out using R 2.11 software and the package “nlme” for GLS models [55].

## Results

**Fig 2:**
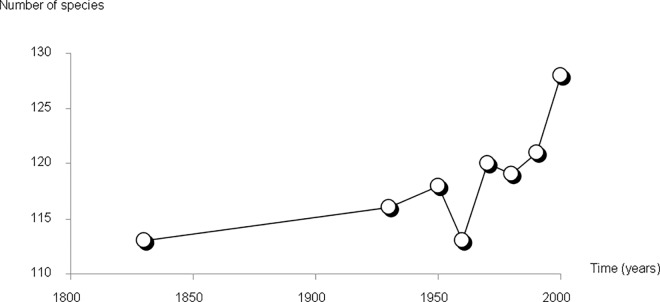
Change in the number of recorded breeding bird species in the Camargue over time.

**Fig 3:**
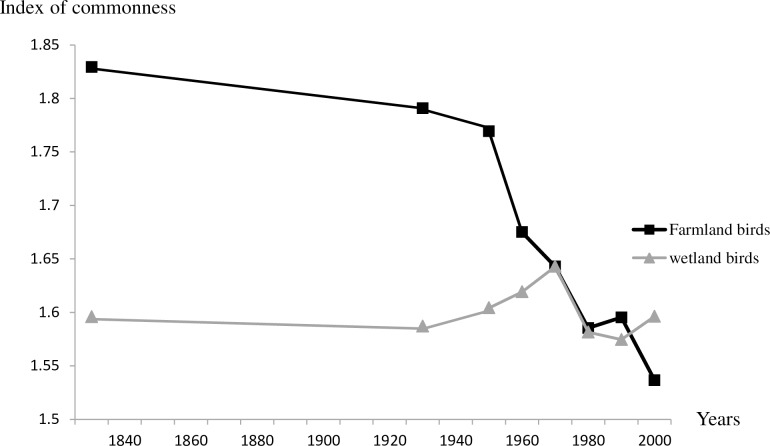
Change in the Community Commonness Index (CCI) of Camargue breeding birds over time. CCI are represented by white circles whereas simulated values of CCI calculated with random samples of species are represented by black squares and their associated standard errors (very small and not visible). The index is estimated by Σa_*i*_/Σ *i*, where *a* designates the abundance of species *i* in the bird community (note that *a* = 0 if the species is absent, 1 if rare, or 2 if common).

**Fig 4:**
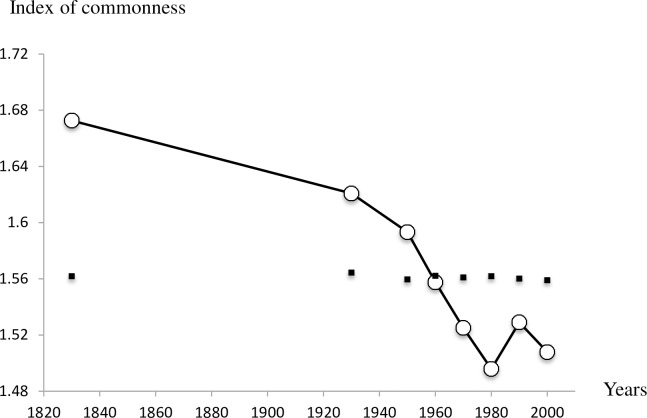
Change in the Community Commonness Index (CCI) of Camargue breeding birds over time, according to their habitat preferences. The index is estimated by Σa_*i*_/Σ *i*, where *a* designates the abundance of species *i* in the bird community (note that *a* = 0 if the species is absent, 1 if rare, or 2 if common).

A marked increase is observed in the number of species recorded in the Camargue breeding avifauna (linear model with temporal autocorrelation, slope: 0.06 ± 0.02 SE; F_1,1_ = 5.59, P = 0.05). This increase seems to have accelerated during the last periods (1960–2009; Fig 2). Conversely, the observed CCI fell over the period (Fig 3), from 1.67 in 1830 to 1.51 in the 2000–09 time period (-0.001 ± 0.0001; F_1,1_ = 31.9, P = 0.001). In other words, the number of individuals per species decreased over time on average. The decrease of the CCI was particularly strong between 1950–59 and 1980–89 before stabilizing at a lower level. The simulations show that the shape and magnitude of this index is clearly different from what is expected from pure fluctuations in species numbers. Apart from 1960–69, the observed CCI was respectively higher and then lower than what is expected from the fluctuation in species richness. Farmland birds but not wetland birds contributed to the decrease of CCI (Fig 4).

**Fig 5:**
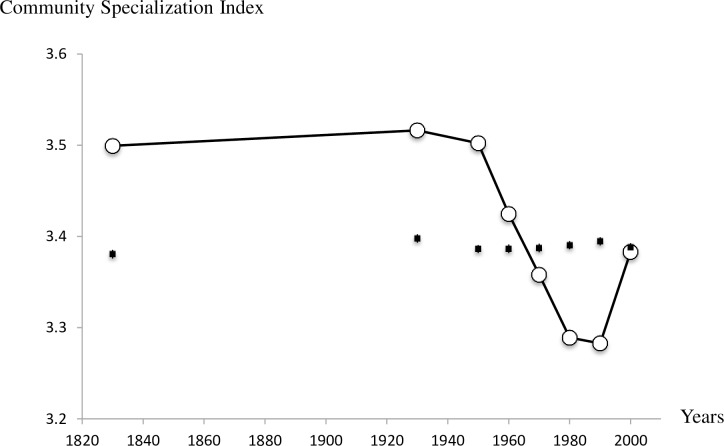
Change in the Community Specialization Index of Camargue breeding birds over time. CSI are represented by white circles whereas simulated values of CSI calculated with random samples of species are represented by black squares and their associated standard errors (very small). The index is estimated by Σ(*a*_*i*_SSI_*i*_)/Σ*a*_*i*_ where *a* is the abundance of species *i* and SSI the species specialization index, a proxy of the level of habitat specialization of each species.

**Fig 6:**
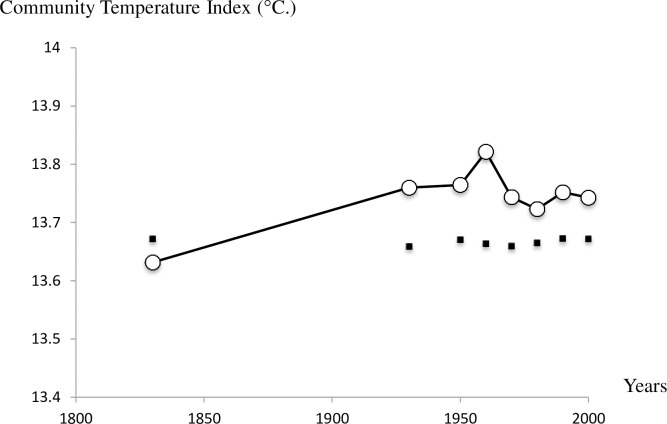
Change in the Community Temperature Index of Camargue breeding birds over time. CTI are represented by white circles whereas simulated values of CTI calculated with random samples of species are represented by black squares and their associated standard errors (very small). The index is estimated by Σ(*a*_*i*_STI_*i*_)/Σ*a*_*i*_ where *a* is the abundance of species *i* and STI the species temperature index, the mean temperature of the area range of each species.

Beyond the species commonness of the pool, the composition of the breeding bird community has also varied over time. 173 species were recorded as breeding during the time periods studied, but only 67 species (or 38.7%) bred in every period. The changes in community composition were also directional as shown by the evolution of CSI and CTI over time. The CSI was stable between 1830 and 1950–59 and then dropped heavily between 1960–69 and 1980–89. There was an increase again in the 2000–09 period; however, this did not reach the pre-1960 levels (Fig 5). The trend in CSI was marginally significant over the entire period (-0.001 ± 0.0001; F_1,1_ = 4.87, P = 0.06) but clearly negative if the trend is estimated from 1930 (-0.003 ± 0.001; F_1,1_ = 9.64, P = 0.02). It can also be noted that as for CCI, the observed values of CSI tend to be above and beyond those expected by a simple fluctuation in species numbers. In other words, the proportion of specialist species (e.g. Penduline Tit, Baillon’s Crake, Red-legged Partridge) in the Camargue bird community declined on average over the period considered and this result cannot be attributed to the trend in species numbers since they were not correlated. The CTI has a slight upward trend (Fig 6) but this is most evident between 1830 (CTI = 13.63°C) and 1930 (CTI = 13.76°C), as the index remained stable between 1930 and 2000–09 (CTI = 13.74°C). The proportion of warm-dwelling birds has increased in the community between the nineteenth century and the first part of the twentieth century but not since. The overall trend therefore only detects a marginal increase (-0.0006 ± 0.0002; F_1,1_ = 5.1, P = 0.06). However, simulations show that observed yearly values of CTI are not significantly different from what is expected, but observed values tend to be consistently higher than expected after 1930.

## Discussion

In this paper, we used field notes and ornithological literature as a proxy for semi-quantitative reconstruction of the past structure and composition of the Camargue bird species pool and their dynamics since 1830.

### Highlighting hidden changes in bird community

Species diversity can mask diverging trends in a species community, and the factors driving the biodiversity decline at a global level while can simultaneously drive increases in biodiversity at the local scale [56]. The construction of a breeding bird matrix associated with a multi-indices approach enabled us to uncover hidden trends in the bird community of the Camargue. In doing so, we showed that species richness has increased over time while community commonness and specialization have decreased. The simultaneous decline in both CSI and CCI suggests that habitat loss and degradation is not only responsible for the decreasing representation of habitat-specialists in the community but also for a decrease in the number of individuals. Moreover, these trends mainly concern farmland birds suggesting that local changes in farmland and/or farming practices were particularly harmful for biodiversity.

According to our results, the main changes in bird assemblages have occurred between the 1950–59 and 1980–89 time periods, coinciding with a time where the landscape of the Camargue faced major changes [57,58]. Aerial photographs show that the surface area of artificial habitats, including cultivated land, salt production and industrial areas, has more than doubled in the Camargue between 1942 and 1984. This increase was strongest between 1953 and 1976, which correspond to the years of strong economic growth in France [58]. Artificial habitats replace natural and semi-natural habitats (Table 1) with a conversion rate particularly high for dry grasslands, salt steppes and temporary marshes whose surface area was reduced by 60% [58]. These habitats were previously used for extensive sheep farming, an activity that collapsed from the 1970s. During this time, farmers of the Camargue largely turned to cereal production (mostly rice farming) marginalizing permanent crops (vineyards and orchards) in the delta. This shift to cereal cropping was accompanied by the expansion of agricultural plot size and the uprooting of hedgerows as in many other areas throughout Europe. Land conversion moving towards an intensive agricultural system is known to be particularly harmful for farmland biodiversity including birds [59]. Locally, we can observe a severe decline (e.g. Little Owl, Red-legged Partridge) or even extinction (e.g. Woodchat Shrike, Scops Owl, Calandra Lark) of several farmland specialists maintained in less-transformed nearby areas like the Crau plain and the Alpilles hills [60,61]. This change is most likely linked with the profound transformation of the agricultural landscape and generalized use of pesticides [62].

**Table 1:** Annual rate of change of surface area in natural habitats of the Camargue.

	Annual rate of change of surface area in natural habitats
1942–53	-0.72
1953–76	-1.48
1976–84	-0.47
1991–2011	+0.1

Loss and gain of natural habitats were estimated through aerial photographs [58] for the 1942–1984 period and by the Regional Nature Park of the Camargue for the 1991–2011 period [63]. Natural habitats include forests, grasslands, dunes, marshes and coastal lagoons.

Changes in landscape also resulted in a diversification of habitats present in the Rhone Delta which probably permitted an increase in species diversity thanks to the emergence of new niches [47]. However, the increase in species richness might be due to the under-detection of rare species in earlier time periods. In return, the decrease in surface area of most habitats led to a general drop in the number of individuals per species as suggested by the simultaneous decline in both CCI and CSI. Apparently, the expansion of human-made and primarily agricultural areas provided insufficient resources for the maintenance or installation of large numbers of bird populations. The artificialization of the hydrological cycle is another process that could explain the different fate suffered by farmland and wetland birds. While the surface area covered with saltpans and rice fields dramatically increased, many marshes were increasingly managed for hunting purposes from the 1960s [58,64,65]. These developing activities involved flooding of large areas during the summer, a time of year where wetlands subject to a Mediterranean climate are typically dry. This type of management contributed to the long-term establishment of new wetland species.

The CTI tends to increase between 1830 and 1930 which is in agreement with a marked increase of global annual mean temperatures at the turn of the twentieth century [66]. More surprisingly, the index was stable over the second half of the twentieth century, despite a significant increase in temperatures in Southern France [67]. This result suggests that the Camargue bird community might have reacted similarly to what is observed elsewhere in Europe [68]. It is, however, possible that the bird community was modified, but the lack of precision of our abundance data makes changes hard to detect. To highlight finer changes in community composition driven by climate change would require more precise data. Climate change responses might also have interacted with confounding factors like land-use change [69]. Most severely reduced habitats surfaces concern dry grasslands and temporary marshes which are particularly represented in the Mediterranean climate. Specialist species from these habitats which have declined (e.g. Calandra Lark, Greater Short-toed Lark, Eurasian Stone-curlew) additionally have affinity for high temperatures and are thus characterized by a high STI. By contrast the expansion of cultivated areas has benefited common bird species of Central and Northern Europe and characterized by lower STI, ultimately leading to the stability of CTI. More empirical data on each species’ sensitivity to temperature changes or specific land-use changes should help to refine these findings.

### Advantages of using historical naturalist data

The benefits of the use of historical naturalist data are twofold. First, they allow the establishment of a point of reference prior to the recent anthropogenic transformation of the landscape. In Western Europe, large-scale wetland conversion started a long time ago: massive loss in alluvial plains were reported in the nineteenth century [70,71], and the largest areas of marshes were already drained between 1705 and 1820 along the French Atlantic coast [72]. Most estuarine and near-shore coastal habitats were severely degraded or driven to virtual extinction well before 1900 [73]. The natural functioning of the Rhone delta was lost from 1868 when the river and seashore were entirely enclosed by dykes. Habitat loss and degradation is thus a long-term process which impacted biodiversity well-before the spread of population monitoring schemes. Second, providing that checklists are available, naturalist data allow us to consider the entire species community. At a time when large areas of natural and semi-natural habitats were lost in the Camargue (1950s-1980s), less than twenty bird species were monitored during the breeding season [74]. Long-term monitoring programs first focused on colonial waterbirds–the Greater Flamingo from 1948, gulls, terns and waders from 1956, and herons from 1964 –for which the Camargue was identified as one of the main nesting areas in Western Europe. Partly due to conservation measures, most of these species have shown a dramatic increase in numbers, a trend that is not confined to the Camargue, but exists throughout most of Europe and the Mediterranean basin [75]. The improving status of few well-monitored and iconic bird species may lead us to conclude that there is a global positive trend in biodiversity in the Camargue [76] and at a broader scale [77]. However, our approach suggests that spectacular increases recorded in some colonial waterbirds could not be generalized to the entire bird community [78], as a majority of species actually experienced a decline.

Data based on birdwatcher’s notes, however, lack important information on sampling effort which can make interpretation of the data difficult and potentially misleading. Birdwatching intensity and our understanding of bird ecology have simultaneously increased over time. A risk of modern birdwatching is a potential bias towards rarer species. An over-representation of “rare” species in recent time can lead us to infer a decrease in average species abundance and specialization or an increase in species richness which are not true. We believe this is not the case in our results show that change in sampling effort was not biased towards species with lower specialization, commonness or temperature preference (S1 and S2 Figs). We therefore think that most of the results cannot be attributed to this potential bias, but reflect true change in community composition. Moreover, the observed trends were generally different from what could be obtained from richness fluctuation. We therefore suggest that any biases present are more likely to lead to under-estimates of the observed changes due to the lack of sensitivity of semi-quantitative estimates.

### Implications for conservation

Interestingly, a recent study has suggested that while rare bird species are now increasing due to specific efforts to improve their conservation status, more common species are declining rapidly due to continuous exposure to global change pressures [79]. Our results suggest that such reconfiguration of community composition should be investigated over longer time periods. A fifth of the Camargue surface area was already benefiting from a strong protection status in the 1950–1980 period; this is much higher than the world average, even when compared with the modern coverage of protected areas [80]. In 1970, half of the delta was declared a Regional Nature Park, a form of protection aiming at reconciling economic development and biodiversity conservation. These measures were nevertheless insufficient to prevent the loss of natural habitats, resulting in the degradation of the remarkable avifauna found in the Camargue.

Populations of colonial waterbirds withconservation priority prospered as a result of intensive management and protection of colonies at local and European levels [81]. However, maps of relative abundance reveal that Camargue is a national stronghold for several marshland and farmland bird species otherwise in sharp decline all over Europe: Yellow Wagtail, Reed Bunting, Western Marsh Harrier, Crested Lark, European Bee-eater, Tree Sparrow, Tawny Pipit, European Roller, Reed Warbler (http://vigienature.mnhn.fr/page/especes-d). These bird species mainly depend on semi-natural habitats which can still be found in local farmland areas of the Camargue devoted to extensive livestock production. According to historical literature, the status of some of these species has clearly deteriorated in the Camargue since the mid-twentieth century. It is therefore crucial to improve bird carrying capacity of the habitats found in the delta by encouraging the adoption of biodiversity-friendly practices by farmers, and promoting natural and semi-natural habitats more favorable to specialist species and able to sustain larger population sizes. To date, agri-environmental schemes (AES) have met limited success with local farmers [57,82]. Recently, there have been initiatives for restoring natural habitats in some protected areas of the Camargue including the restoration of marshes and grasslands on abandoned rice fields and salt pans [83,84].

How restoration projects can help in improving the composition and structure of the bird community remains to be tested. Future orientations of the Common Agricultural Policy will certainly have a major impact on bird populations in the Camargue, with possible positive effects if traditional extensive livestock farming is favored by European aids. Interestingly, the recent increase in CSI corresponds to a slight increase in natural areas in the Camargue (Table 1) due to the evolution of 3500 hectares of abandoned cultivated fields towards natural meadows and marshlands.

## Conclusion

The use of historical data can be useful for measuring biodiversity trends. The measured indices highlighted profound changes in the structure and composition of the breeding bird community of the Rhone Delta with a trend of fewer individuals per species and a decreased representation of habitat-specialist species in the community. These modifications mainly occurred between the 1950s and 1980s, coinciding with the massive loss in surface of natural habitats. Political choices for the economical development of the area right following the Second World War were the main drivers of land-use change with large investments for the spread and intensification of cereal farming. These important changes in the bird community could not have been revealed by existing monitoring schemes which were initiated after the changes in the Camargue landscape and chiefly focus on emblematic colonial waterbirds which are in a better state of conservation. The Camargue is not a unique case as large-scale land-cover planning and agriculture intensification happened simultaneously in many regions of Western Europe. We can fear that the loss of common birds—especially farmland birds—observed for 20 or 30 years in Europe is only the continuation of an older and of greater decline [19,85]. More generally, our results provide empirical evidence for the so-called “shifting baseline” syndrome whereby reference population sizes used to evaluate changes tend to decrease over time given the lack of appropriate data to document past situations [86,87]. To better understand biodiversity dynamics, managers need to access historical data so that more relevant and informed decisions about recovery and restoration targets can be proposed [88]. Reconstructing estimates of past population sizes and community composition responds to a need for a well-defined reference state ecosystem to evaluate changes and restoration success [72].

## Supporting Information

S1 Fig Change in Community Specialization Index of common breeding birds in the Camargue over time. Only species with an abundance coded by 2 (“common”) were included in this analysis.
(TIF)

S2 Fig Change in species richness of the breeding bird community in the Camargue over time. Only common species, coded by 2 in the abundance matrix were included.
(TIF)

S1 Table Breeding bird species in the Camargue with annotated abundance over time. Abundance is given by a species commonness index coded by “0” if breeding was unknown at that time period, “1” if the species was rare, and “2” if the species was common.
(XLS)

S2 Table Main habitat used by breeding birds in the Camargue. Our classification is based on the European Bird Census Council (EBCC) classification for the Mediterranean region with an additional category (wetland) and additional species not assessed yet by the EBCC experts.
(XLSX)
