# Supplementary figures and images for: When Common Birds Became Rare: Historical Records Shed Light on Long-Term Responses of Bird Communities to Global Change in the Largest Wetland of France
Source: PLoS One. 2016 Nov 10;11(11):e0165542. doi: 10.1371/journal.pone.0165542 (PMC5104452; doi:10.1371/journal.pone.0165542)

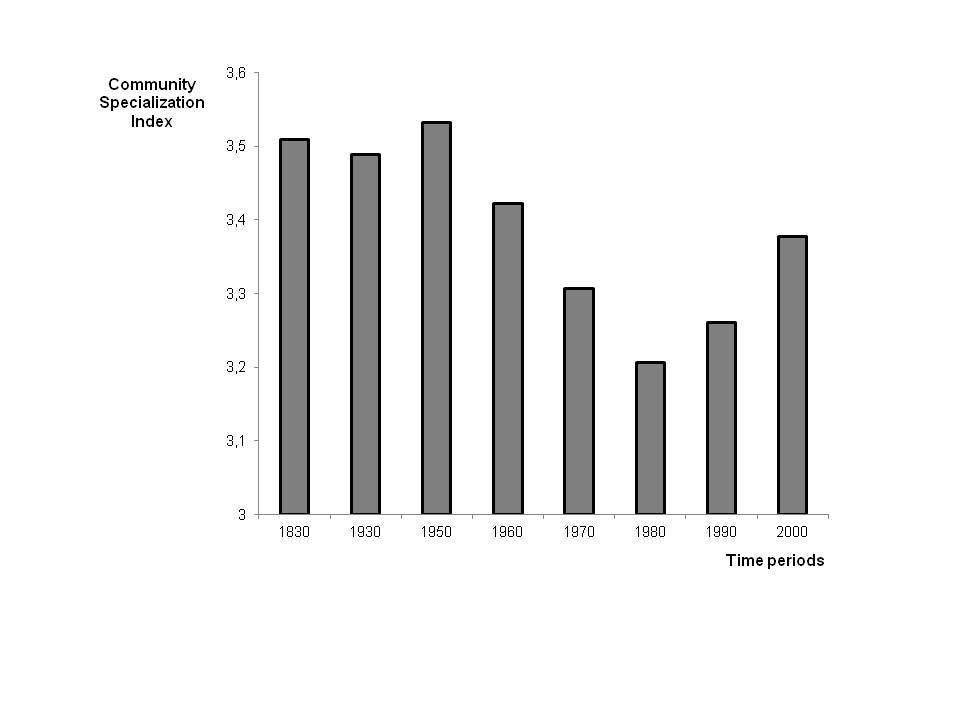

Supplement: S1 Fig — Only species with an abundance coded by 2 (“common”) were included in this analysis. (TIF) [file pone.0165542.s001.tif]

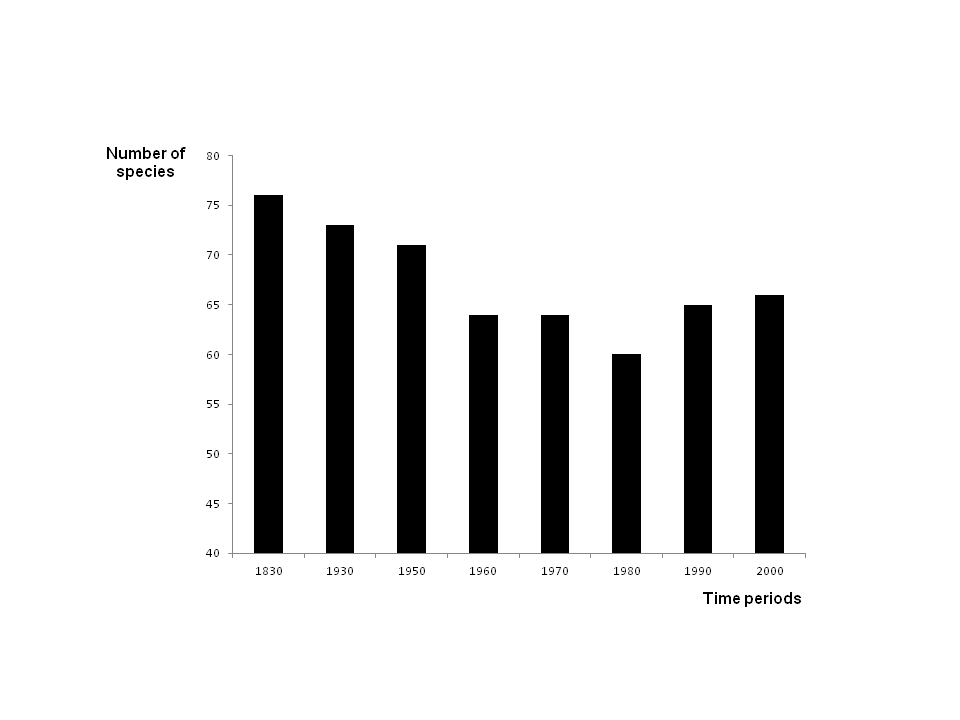

Supplement: S2 Fig — Only common species, coded by 2 in the abundance matrix were included. (TIF) [file pone.0165542.s002.tif]
